# Advanced T-cell Lymphoma with Penile Involvement Revealed by ^18^ F-FDG PET/CT: A Rare Presentation

**DOI:** 10.1055/s-0045-1810100

**Published:** 2025-07-18

**Authors:** Yassir Benameur, Adnane Hammani, Jaafar El Bakkali, Abderrahim Doudouh

**Affiliations:** 1 Department of Nuclear Medicine, Mohammed V Military Teaching Hospital, Faculty of Medicine and Pharmacy, Mohammed V University of Rabat, Rabat, Morocco; 2 Department of Clinical Haematology, Mohammed V Military Teaching Hospital, Faculty of Medicine and Pharmacy, Mohammed V University of Rabat, Rabat, Morocco

**Keywords:** ^18^ F-FDG, aggressive lymphomas, penile involvement, T-cell lymphomas

## Abstract

T-cell lymphomas are rare malignancies that frequently involve extranodal sites, but penile localization remains exceedingly uncommon. We report the case of a 21-year-old man diagnosed with cutaneous T-cell lymphoma who underwent 18F-fluorodeoxyglucose positron emission tomography/computed tomography ( ^18^ F-FDG PET/CT) for initial staging. The scan revealed widespread nodal and extranodal disease, including unexpected intense fluorodeoxyglucose uptake in the corpora cavernosa, in the absence of penile symptoms. Penile involvement in lymphoma, whether primary or secondary, Hodgkin or non-Hodgkin, is exceptionally rare, with very few cases reported—most involving B cell histologies. This finding underscores the utility of PET/CT in identifying clinically silent and atypical disease sites in aggressive lymphomas such as peripheral T-cell lymphoma. The extent of dissemination, including renal, adrenal, bone marrow, and penile involvement, reflects a high tumor burden and portends a poor prognosis. Early recognition of such patterns through PET/CT is essential for appropriate therapeutic planning and may impact patient outcomes.

## Introduction

T-cell lymphomas are uncommon and heterogeneous malignancies, accounting for approximately 10 to 15% of all non-Hodgkin lymphomas. 1 They often present with cutaneous, nodal, and visceral involvement. Penile localization is exceedingly rare and sparsely documented in the literature. We report the case of a young adult diagnosed with aggressive cutaneous T-cell lymphoma, presenting with widespread disease, including involvement of the corpora cavernosa, as revealed by 18F-fluorodeoxyglucose positron emission tomography/computed tomography ( ^18^ F-FDG PET/CT) during initial staging.

## Case Presentation

**Fig. 1:**
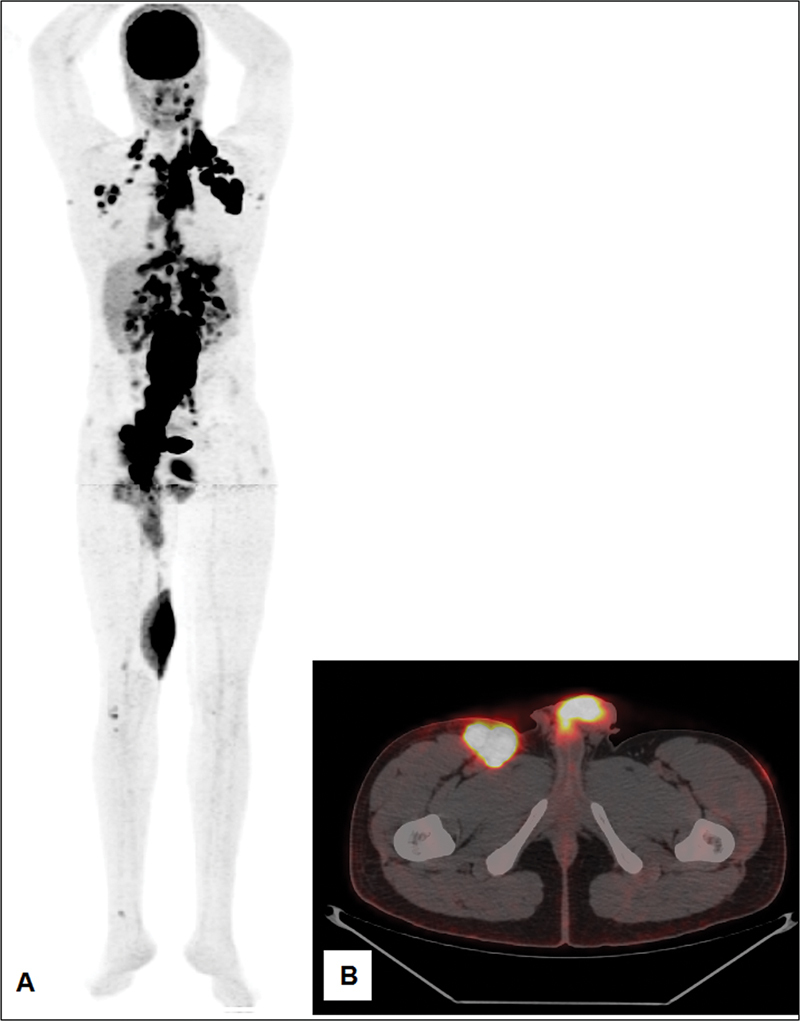
( **A** ) Maximum intensity projection (MIP) image showing multiple intensely hypermetabolic lesions involving supra- and infradiaphragmatic lymph nodes, as well as several extranodal sites: stomach, left kidney, bilateral adrenal glands, penis (corpora cavernosa), intramedullary spine at L1–L2, and subcutaneous tissue of the right thigh. ( **B** ) Axial PET/CT fusion image confirming hypermetabolic penile involvement. PET/CT, positron emission tomography/computed tomography.

A 21-year-old male with no significant past medical history presented with pruritic cutaneous lesions and progressive cervical lymphadenopathy. Clinical examination revealed erythematous infiltrated plaques on the trunk and upper limbs, along with palpable lymph nodes in the cervical and axillary regions. A skin biopsy confirmed the diagnosis of cutaneous T-cell lymphoma. The patient was referred to our department for whole-body ^18^ F-FDG PET/CT to assess disease extent. The scan was performed 60 minutes after injection of 240 MBq of ^18^ F-FDG, and revealed widespread lymphadenopathy involving cervical, mediastinal, axillary, abdominal, pelvic, and inguinal regions. In addition, numerous extra-nodal hypermetabolic lesions were noted, including involvement of the stomach, left kidney, adrenal glands, bone marrow, and subcutaneous tissue of the right thigh. Unexpectedly, the ^18^ F-FDG PET/CT also showed increased metabolic activity in the penis, particularly in the region of the corpora cavernosa, without any corresponding clinical symptoms or local findings ( Fig. 1 ). This suggested asymptomatic penile involvement, which is extremely rare in lymphoma. No significant metabolic activity was detected in the liver, spleen, or pancreas.

## Discussion

Peripheral T-cell lymphomas (PTCLs) are a heterogeneous and generally aggressive group of non-Hodgkin lymphomas that frequently exhibit extranodal involvement. Common extranodal sites include the skin, gastrointestinal tract, bone marrow, and liver. 2 Among these, cutaneous T-cell lymphomas such as mycosis fungoides and Sézary syndrome represent a more indolent end of the spectrum, but transformation into aggressive variants can occur and is associated with widespread dissemination. 3 Penile involvement in lymphoma, whether primary or secondary, Hodgkin or non-Hodgkin, is exceedingly rare, and particularly uncommon in PTCL. Most reported cases involve B cell lymphomas. 4 5 According to the largest review available, lymphomas of the male genital tract represent less than 5% of all extranodal lymphomas, with the testes being the most frequently affected site. Penile localization is exceptional, with only two cases reported in the literature, one primary and one secondary. 6 The pathophysiology underlying such an unusual localization remains speculative. Proposed mechanisms include retrograde lymphatic spread from pelvic or inguinal lymph nodes, hematogenous dissemination, particularly in aggressive subtypes, and direct extension from adjacent lymphatic structures. 7 In the present case, the absence of penile symptoms despite clear metabolic activity involving the corpora cavernosa underscores the value of ^18^ F-FDG PET/CT in identifying clinically silent disease. This imaging modality has become essential in the staging and therapeutic monitoring of aggressive lymphomas, especially PTCLs, where the extent of disease often surpasses clinical or conventional radiological findings. 8 9 The extensive disease burden observed here, including renal, adrenal, gastric, bone marrow, subcutaneous, and penile involvement, indicates a highly disseminated and biologically aggressive form of systemic T-cell lymphoma. This pattern necessitates prompt initiation of systemic multi-agent chemotherapy and, in some cases, consideration of consolidation with hematopoietic stem cell transplantation depending on treatment response and histologic subtype. 10 PTCLs are aggressive malignancies characterized by poor prognosis, particularly in cases with extensive extranodal involvement. The 5-year overall survival rate is approximately 30%. 11 The presence of multiple extranodal sites, such as the kidneys, adrenal glands, bone marrow, and penis, as observed in this case, is associated with a higher tumor burden and correlates with poorer outcomes. 11 Early and accurate staging using ^18^ F-FDG PET/CT is crucial for guiding treatment decisions and improving survival chances. Despite advances in therapy, PTCL patients often require intensive systemic treatment and may benefit from inclusion in clinical trials evaluating novel agents and targeted therapies. 12

## Conclusion

This case highlights a rare and aggressive presentation of T-cell lymphoma with penile involvement, incidentally revealed by PET/CT imaging. It underscores the indispensable role of ^18^ F-FDG PET/CT in staging aggressive lymphomas, particularly for detecting atypical and clinically silent disease sites. Although penile involvement did not alter therapeutic planning or prognostic assessment in this case due to the already widespread disease, it represents a potential imaging pitfall that clinicians should be aware of during interpretation.

## References

[1] Vose J M Peripheral T-cell non-Hodgkin's lymphoma Hematol Oncol Clin North Am 2008 22 05 997 1005, x 18954748 10.1016/j.hoc.2008.07.010

[2] International T-Cell Lymphoma Project Vose J Armitage J Weisenburger D International peripheral T-cell and natural killer/T-cell lymphoma study: pathology findings and clinical outcomes J Clin Oncol 2008 26 25 4124 4130 18626005 10.1200/JCO.2008.16.4558

[3] Willemze R Jaffe E S Burg G WHO-EORTC classification for cutaneous lymphomas Blood 2005 105 10 3768 3785 15692063 10.1182/blood-2004-09-3502

[4] Arambulo S Calle A Vela J M Sotelo M J Advanced penile lymphoma: case report and review of the literature J Cancer Res Ther 2023 19 03 823 825 37470619 10.4103/jcrt.jcrt_593_21

[5] Diao L Yang S Shang P Hou Z Report of penis lymphoma and review of the literature Asian J Surg 2022 45 11 2528 2529 35717302 10.1016/j.asjsur.2022.05.136

[6] Schniederjan S D Osunkoya A O Lymphoid neoplasms of the urinary tract and male genital organs: a clinicopathological study of 40 cases Mod Pathol 2009 22 08 1057 1065 19377442 10.1038/modpathol.2009.65

[7] Nakayama F Sheth S Caskey C I Hamper U M Penile metastasis from prostate cancer: diagnosis with sonography J Ultrasound Med 1997 16 11 751 753 9360239 10.7863/jum.1997.16.11.751

[8] Naik N Lin M Lin P Genitourinary involvement of lymphomas on FDG-PET Br J Radiol 2018 91(1086):2.0170273E7 10.1259/bjr.20170273 PMC6223267 29322833

[9] Feeney J Horwitz S Gönen M Schöder H Characterization of T-cell lymphomas by FDG PET/CT AJR Am J Roentgenol 2010 195 02 333 340 20651187 10.2214/AJR.09.3665

[10] El-Galaly T C Villa D Gormsen L C Baech J Lo A Cheah C Y FDG-PET/CT in the management of lymphomas: current status and future directions J Intern Med 2018 284 04 358 376 29989234 10.1111/joim.12813

[11] Savage K J Peripheral T-cell lymphomas Blood Rev 2007 21 04 201 216 17512649 10.1016/j.blre.2007.03.001

[12] Luan Y Li X Luan Y Therapeutic challenges in peripheral T-cell lymphoma Mol Cancer 2024 23 01 2 38178117 10.1186/s12943-023-01904-w PMC10765866

